# A Novel Theoretical Hypothesis on Comorbidity in the Elderly—The Disease of Sympathetic Overdrive (DSO)

**DOI:** 10.1002/agm2.70026

**Published:** 2025-07-09

**Authors:** Li Jie, Kong Jian, Yu Boxin, Li Hongyang

**Affiliations:** ^1^ Department of Elderly and Cadrethe First Hospital of Jilin University Changchun China; ^2^ Department of General Practice First Hospital of Jilin University Changchun China

**Keywords:** comorbidity, geriatrics, hypothesis, sympathetic overdrive

## Abstract

The coexistence of multimorbidity and geriatric syndromes is a big character and role target to manage for the clinic works in older age, but related theories still require innovation and exploration. The disease of sympathetic overdrive (DSO) is a new hypothesis, a disease full of characteristics of organics and emotion, multiple systems, and lifestyle illness. The hypothesis of DSO might be able to explain and improve the theory about the coexistence of multimorbidity and geriatric syndromes and even more!.

## Graphical abstract

A novel hypothesis: The disease of sympathetic overdrive (DSO) as a unifying mechanism for comorbidity in the elderly.

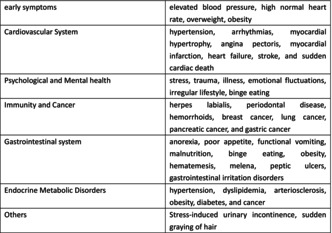

## 1 Introduction

The main features of geriatrics are geriatric comorbidity and geriatric syndromes. Many related problems often occur simultaneously in the same individual, affecting older people's physical health and quality of life. The question arises as to whether these two situations are merely coincidental overlaps or whether there is a common underlying pathophysiological mechanism. Answering this question can advance our understanding of the occurrence of geriatric comorbidity and geriatric syndromes, and promote the development of relevant treatment and management strategies.

The sympathetic nervous system is the principal component of the autonomic nerve and is mostly distributed in organs such as the gastrointestinal system, heart, resistance blood vessels, kidneys, and skin. The catecholamines released by its terminals can impact the immune cells' functions, insulin and insulin sensitivity, as well as electrolyte balance, etc. Thus, the dysfunction within the sympathetic nervous system may lead to complex functional abnormalities or disorders of multiple systems, tissues, and organs. These multiple system dysfunctions caused by the disorder or excessive activation of the sympathetic nerve should fall under the unified category of disorder of sympathetic overdrive (DSO). Currently, the academic community has provided numerous fragmented explanations for various diseases stemming from sympathetic nervous system dysfunction. Still, it has yet to approach the significant issues arising from these multisystem functional abnormalities from an independent and systematic perspective, nor has it developed research and preventive strategies grounded in this understanding. DSO may represent a long‐overlooked yet fundamentally relevant disease, both ancient and novel. It could potentially be a new theoretical hypothesis for geriatric comorbidities and geriatric syndromes. This paper aims to discuss the definition of the disease, key clinical symptoms of DSO, risk factors, primary mechanisms, prevention and control strategies, and their implications.

## 2 Disease Definition and The Disease of Sympathetic Overdrive

The nomenclature of any disease should align with the basic elements, including clinical signs and symptoms, risk factors, mechanisms, adverse outcomes, or endpoints.

The dysfunction of the sympathetic nervous system has historically been described and analyzed in a scattered manner concerning various diseases' onset and progression, without being comprehensively recognized, studied, named, or managed as a distinct entity. Our research group first proposed the hypothesis of the DSO within the academic community [1], and DSO meets the basic elements of the disease. Its characteristics include complex clinical symptoms, numerous risk factors, and poor prognoses, with sympathetic overdrive as its core component, along with various other related mechanisms.

## 3 Symptoms and Classification of DSO

DSO‐related symptoms are numerous, complex, and dispersed across various systems and organs, summarized as follows (Table 1):

**TABLE 1:** Classification of DSO.

Early symptoms	Elevated blood pressure, high normal heart rate, overweight, obesity
Cardiovascular system	Hypertension, arrhythmias, myocardial hypertrophy, angina pectoris, myocardial infarction, heart failure, stroke, and sudden cardiac death
Psychological and mental health	Stress, trauma, illness, emotional fluctuations, irregular lifestyle, binge eating
Immunity and cancer	Herpes labialis, periodontal disease, hemorrhoids, breast cancer, lung cancer, pancreatic cancer, and gastric cancer
Gastrointestinal system	Anorexia, poor appetite, functional vomiting, malnutrition, binge eating, obesity, hematemesis, melena, peptic ulcers, gastrointestinal irritation disorders
Endocrine metabolic disorders	Hypertension, dyslipidemia, arteriosclerosis, obesity, diabetes, and cancer
Others	Stress‐induced urinary incontinence, sudden graying of hair

### 3.1 Early Symptoms

There may be many people who experience a short period, mild overdrive of the sympathetic nerve, which is not a symptom of DSO, such as temporary dizziness, palpitations, body tremors, hyperhidrosis, etc.

Typical early symptoms associated with DSO include elevated blood pressure [2], high normal heart rate [3, 4], overweight and obesity, etc., which are currently recognized by the academic community as having significant pathological implications. For example, the adjustment of hypertension diagnosis and control standards in recent years suggests that normal high blood pressure is very important for the early management of hypertension. The latest ESC Guidelines management has identified blood pressure exceeding 120/70 mmHg as hypertension [2], marking a recent advancement. Both high normal heart rate [4] and obesity can be associated with adverse endpoint events, which has attracted academic attention.

### 3.2 Cardiovascular System

The sympathetic nervous system is widely distributed throughout the heart and blood vessels, playing a key role in regulating heart rate, blood pressure, myocardial contraction, and blood volume [5]. Prolonged and irregular excessive activation may lead to a variety of cardiovascular diseases [6, 7], such as hypertension, arrhythmias, myocardial hypertrophy, angina pectoris, myocardial infarction, heart failure, stroke, and sudden cardiac death, posing a significant threat to public health and life.

### 3.3 Psychological and Mental Health

Among populations, especially the elderly and the period of the COVID‐19 pandemic [8]. Factors such as stress, trauma, illness, emotional fluctuations, irregular lifestyle, and binge eating activate the sympathetic nervous system through various mechanisms, leading to psychological symptoms such as insomnia, depression [9, 10], anxiety [11], and may trigger chronic pain, weakness, malnutrition, polypharmacy, and geriatric syndromes. Severe cases can lead to hypertension, acute coronary syndrome, stroke, sudden death, significantly impacting the quality of life of patients or posing life‐threatening risks.

### 3.4 Immunity and Cancer

The occurrence and progression of numerous infections and malignant tumors [12] are significantly influenced by psychological factors, such as herpes labialis [13], periodontal disease, hemorrhoids, breast cancer, lung cancer, pancreatic cancer, and gastric cancer [14]; The fundamental issues involve psychological stressors such as pressure, anxiety, and depression, which lead to the overdrive of the sympathetic [15], affecting various immune active cells in the bone marrow and lymphatic system, suppressing the immune system function, and triggering the aforementioned infections and malignant tumors.

The regulation between mental health and immune system function is indispensable for the sympathetic nervous system. According to the DSO hypothesis, actively engaging in psychological interventions, coupled with the administration of beta‐blocker [16], may present a crucial strategy for improving prognosis and quality of life in these patients. Clinical studies have already reported successful prolongation of survival and improvement of survival rates in breast cancer, lung cancer, and thyroid cancer by psychological counseling and beta‐blocker application.

### 3.5 The Gastrointestinal System

The sympathetic nervous system, as a primary component of the autonomic nervous system, plays a crucial role in the regulation of digestive functions [17, 18]. Influenced by various environmental, psychological factors, and ailments; mild to moderate sympathetic overactivity can lead to symptoms such as anorexia, poor appetite, functional vomiting, malnutrition, binge eating, and obesity. Severe sympathetic hyperactivation can result in hematemesis, melena, certain types of peptic ulcers, gastrointestinal irritation disorders [19], and can even pose a fatal risk.

### 3.6 Endocrine Metabolic Disorders

Emotional responses, visual stimuli, and the activation of sensory organs can significantly excite the central nervous system, leading to excessive activation of the sympathetic nervous system, which in turn affects the sensitivity of insulin and insulin receptors [19]. This dynamic plays a crucial role in regulating the secretion of digestive fluids, blood glucose levels, and blood volume. Vivid representations such as craving for food upon sight, the sensation of hunger triggered by seeing food, and salivation illustrate the functional changes in the digestive and endocrine systems resulting from excessive sympathetic nervous system activation. The prevalence of binge eating, irregular dietary habits, and consumption of stimulants within the population often leads to frequent, irregular, and intense sympathetic overactivity, contributing to various endocrine metabolic diseases, including hypertension, dyslipidemia, arteriosclerosis, obesity [20, 21], diabetes [22], and cancer. Thus, according to the DSO hypothesis, unhealthy lifestyle choices are fundamentally linked to the pathophysiological mechanisms underlying the emergence and progression of these widely recognized societal health issues, primarily driven by excessive sympathetic nervous system activation.

### 3.7 Others

Stress‐induced urinary incontinence [23] and sudden graying of hair [24] are closely linked to short‐term elevated tension, pressure, and anxiety, with excessive activation of the sympathetic nervous system playing a crucial role. Research has shown that the degeneration and apoptosis of skin melanocytes occur only in environments with high concentrations of norepinephrine [24].

The clinical characteristics and current status of DSO present the following features: coexistence of psychological disorders and somatic diseases; predominance of metabolic syndrome and lifestyle‐related diseases; a diverse spectrum of advanced diseases with early recognition yet to be clearly defined, while research on mechanisms requires consensus within the academic community.

## 4 The Primary Risk Factors and Classifications of DSO are Extensive and Complex

They include lifestyle factors such as smoking, excessive alcohol consumption, irregular daily routines, binge eating, and lack of physical activity. Social factors encompass issues like overwork, stress, conflict, romantic relationships, and interpersonal interactions. Natural factors consider elements such as environmental conditions and pollution, aging, health management, natural disasters, and the prevalence of diseases.

## 5 Mechanisms

The mechanisms underlying DSO are intricate, it may involve oxidative stress [25], apoptosis [26, 27], inflammatory responses [28, 29], insulin resistance [30, 31], hyperinsulinemia [32, 33, 34], and the activation of the renin‐angiotensin‐aldosterone system [35, 36], potentially resulting in clinical comorbidities across multiple organ systems and geriatric syndromes. Many of the details involved require further investigation. Therefore, studying the mechanisms of DSO and exploring effective therapeutic strategies is crucial for reducing its incidence and improving survival rates.

## 6 Exploration of Treatment Strategies and Methods for DSO

The DSO hypothesis involves redefining, summarizing, understanding, and organizing numerous prevalent clinical conditions, geriatric syndromes, and their risk factors. Its goal is to enhance the theoretical foundation for coordinated and comprehensive prevention and control of related risk factors and diseases within large populations, particularly among the elderly. It emphasizes the importance of fostering a harmonious social and natural environmental context while actively intervening in mental health issues; it advocates for healthy lifestyles and promotes healthy aging.

### 6.1 Key Strategies

Promote the scientific diet advocated by the WHO [37], engage in moderate exercise [38], quit smoking and alcohol, maintain an optimistic outlook, and lead a regular lifestyle; implement management of multiple risk factors throughout the entire life cycle. Utilize beta‐blockers based on individual circumstances [39]; in the intermediate and advanced stages, treatments should be conducted according to specific diseases and conditions of various systems, guided by established protocols.

## 7 DSO Hypothesis Innovation

A comprehensive reevaluation and understanding of the core role of commonly recognized, segregated, and controlled sympathetic nerve dysfunction in the diseases affecting multiple systems has been proposed. This includes the preliminary introduction of the DSO concept and its main clinical manifestations. Additionally, we aim to identify and analyze the social, environmental, lifestyle, and psychological factors that lead to excessive activation of the sympathetic nervous system. This offers new strategies and directions for the early, systematic, and comprehensive prevention and control of many prevalent diseases and geriatric syndromes. The DSO hypothesis requires further validation and refinement. It may represent a longstanding condition, affecting multiple systems and exhibiting a wide range of symptoms. A theoretical framework for systematically understanding, summarizing, and exploring related issues could facilitate healthy aging in populations, significantly enhancing both the theoretical and clinical approaches to preventing and controlling geriatric comorbidities and syndromes.

## Author Contributions

Li Jie: oversight and leadership responsibility for the article; Kong Jian: management activities; Yu Boxin: writing the initial draft; Li Hongyang: Evidence collection.

## Consent

The authors have nothing to report.

## Conflicts of Interest

The authors declare no conflicts of interest.
